# Summary of the International Patient Safety Conference, June 28—29, 2019, Kathmandu, Nepal

**DOI:** 10.1186/s13037-019-0214-4

**Published:** 2019-11-18

**Authors:** Deepak C. Bajracharya, Kshitij Karki, Chhiring Yangjen Lama, Rajesh Dhoj Joshi, Shankar Man Rai, Sudhakar Jayaram, Amit Tomer, John Zervos, Mohammad Imran Khan, Arjun Sapkota, Madan Kumar Upadhyaya, Paul E. Kilgore

**Affiliations:** 1 Group for Technical Assistance, Lalitpur, Nepal; 2 Smith College, Northampton, Massachusetts USA; 3 Kathmandu Model Hospital, Kathmandu, Nepal; 4 Kirtipur Hospital, Kathmandu, Nepal; 5 Nepal Mediciti Hospital, Lalitpur, Nepal; 6 Henry Ford Health System Global Health Initiative (HFHS GHI), Detroit, MI USA; 7 Precision Health Consultants, Karachi, Pakistan; 8 Curative Service Division, Department of Health Services, Ministry of Health and Population, Kathmandu, Nepal; 9 Quality Standard and Regulation Division, Ministry of Health and Population, Kathmandu, Nepal; 10 Wayne State University, Detroit Michigan USA, HFHS GHI, Detroit, MI USA

## Abstract

Globally, medical errors are associated with an estimated $42 billion in costs to healthcare systems. A variety of errors in the delivery of healthcare have been identified by the World Health Organization and it is believed that about 50% of all errors are preventable. Initiatives to improve patient safety are now garnering increased attention across a range of countries in all regions of the world. From June 28--29, 2019, the first *International Patient Safety Conference* (IPSC) was held in Kathmandu, Nepal and attended by over 200 healthcare professionals as well as hospital, government, and non-governmental organization leaders. During the conference, presentations describing the experience with errors in healthcare and solutions to minimize future occurrence of adverse events were presented. Examples of systems implemented to prevent future errors in patient care were also described. A key outcome of this conference was the initiation of conversations and communication among important stakeholders for patient safety. In addition, attendees and dignitaries in attendance all reaffirmed their commitment to furthering actions in hospitals and other healthcare facilities that focus on reducing the risk of harm to patients who receive care in the Nepali healthcare system. This conference provides an important springboard for the development of patient-centered strategies to improve patient safety across a range of patient care environments in public and private sector healthcare institutions.

## Background

Errors and other events in the healthcare facilities that negatively impact the patients lead to extended hospitalizations, death, and increased health care costs. According to World Health Organization (WHO), the costs associated with medication errors alone account for almost 1 % ($42 Billion USD) of the overall expenditure in healthcare worldwide [1]. Moreover, 42.7 million patients experience adverse events in hospitals each year worldwide and about 2.6 million deaths occur every year due to adverse events and unsafe care in lower-middle income countries.

Over the past two decades, there has been increasing awareness of the need to improve the quality of healthcare in all countries, regardless of income or level of development. After the publication of a galvanizing report (To Err is Human) by the Institute of Medicine [2] that addressed patient safety concerns in the US, international organizations such as the WHO as well as multiple governments began working in this front. More recently, since 2016, the WHO has been collaborating with the government of the UK and Germany to host the Annual Global Ministerial Summit on Patient Safety [3].

A wide range of local organizations, hospitals, professional societies, and international organizations are now focused on disseminating and implementing innovative tools and systems that improve patient safety. As part of WHO’s efforts to improve patient safety, a surgical safety checklist to standardize procedures for safer surgical practices has been developed [4]. In addition, the WHO has published a Multi-professional Patient Safety Curriculum Guide to promote patient safety education among medical professionals and educators [1]. WHO has also distributed a safe childbirth checklist, Guidelines on Patient Safety Incident Reporting and Learning Systems, and a Minimum Information Model to improve patient safety culture in health facilities around the globe. In Nepal’s context, conversations surrounding patient safety have started receiving attention only recently. The overarching goal of the International Patient Safety Conference is to bring together key stakeholders and leaders in Nepali healthcare delivery to initiate constructive conversations around improving patient safety and healthcare quality in Nepal.

### Impact of healthcare delivery on patients

Around the world and in Nepal, patients who seek medical care may be seen in clinics, emergency departments or inpatient facilities. In such settings, healthcare personnel are often operating under stressful conditions with limited time for conversation with patient families and the evaluation of individual patients. In this conference, expert panelists noted that healthcare personnel are now working longer hours and have increased demands to be more productive than ever before. That is, they are under increasing pressure to see an ever-greater number of patients. On the other hand, complex hospital settings and systems have made it harder for patients to navigate health facilities and procedures.

**Fig. 1:**
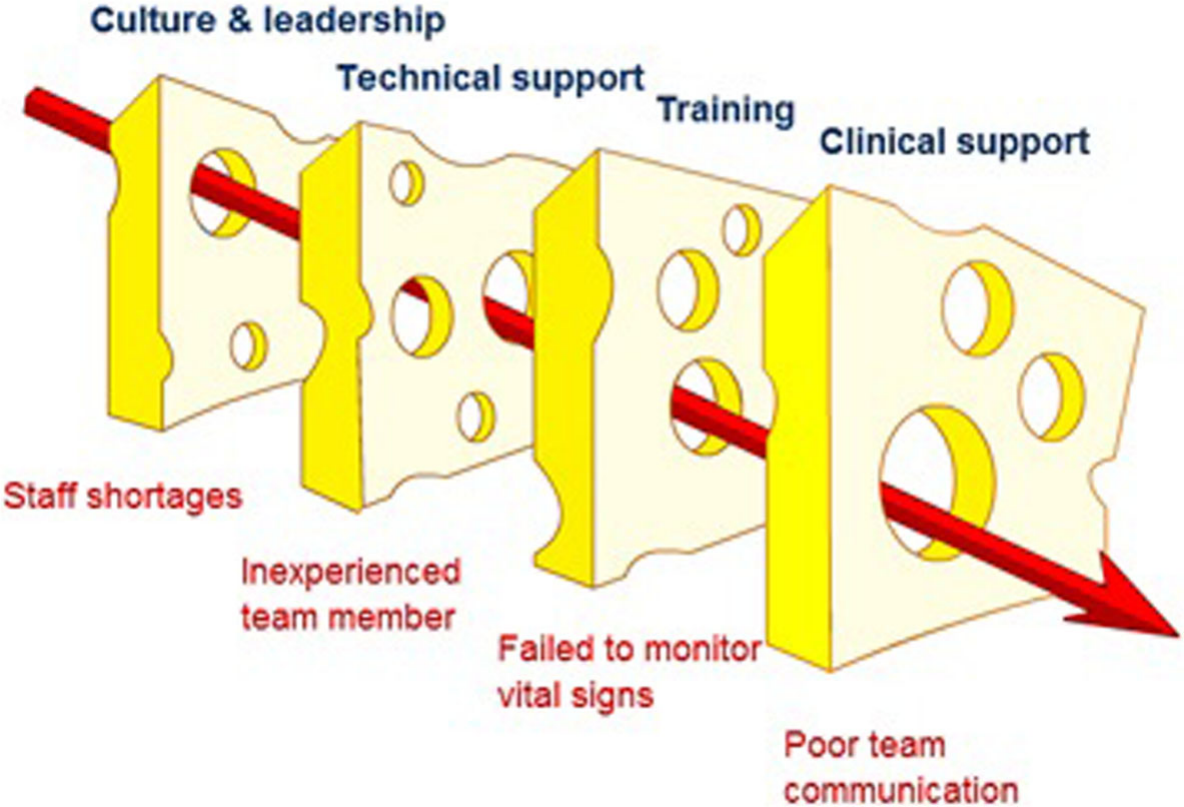
Swiss Cheese Model of how hospital amenities may be penetrated to compromise patient safety [5]

In many hospitals, despite the presence of highly trained and experienced physicians, important details regarding patient histories, medications or procedures completed in the hospital may be incompletely or inaccurately recorded. In such environments, the lack of systematic approaches and checklists that ensure that all the steps for a patient’s care are completed has the potential of putting patients at risk for untoward events such as medication or surgical procedural errors (Fig. 1). In this conference, hospital delegates mentioned that they have been using as well as modifying patient care checklists to fit their institutional service procedures. However, conference delegates noted that the checklists and other recommended processes have not been fully adopted by all health facilities in and outside the Kathmandu Valley.

Conference presenters highlighted the fact that patients often receive care from multiple healthcare professionals during their hospitalization. During their care, there may be little coordination between the doctors involved in an individual patient’s care. The lack of transparent communication between the management and the healthcare staff as well as between senior staff and their subordinates also affect the daily operations of the hospital and quality of care that the patients receive.

### How hospitals and other organizations work to protect patients

A prominent topic featured during the conference presentations and discussions were patient safety checklists developed by the WHO and collaborating organizations. Some of these checklists have been created to standardize high-quality surgical healthcare services. The panelists and delegates at the conference also highlighted the implementation and impact of antimicrobial stewardship programs that are designed to improve the judicious use of antimicrobial therapies (Additional file 1: Figure S1). These programs aim to reduce exposure of patients to unnecessary therapies while also reducing the emergence of multi drug-resistant bacteria in healthcare facilities. Other conference presenters discussed the use of continuous professional development programs as well as the implementation of improved communication methods that help the medical care team in ensuring the continuity of high-quality care among teams that transfer care from one work shift to the next (Additional file 2: Figure S2). In the conference, Nepali government representatives underscored the necessity of developing and implementing minimum service standards in all public and private hospitals in Nepal (Additional file 3: Figure S3). These standards are designed to help raise the quality of medical care, ensure that patients are treated equitably and minimize the risk of harm during their encounter with the healthcare system.

### Key lessons from clinical experience and other industries

From years of clinical experience accrued by conference panelists, several key lessons emerged. First, senior Nepali physicians emphasized the need for rigorous self-examination of clinical errors and patient outcomes resulting from errors. Similarly, attendees advocated for more rigorous regulation of adverse events and errors by hospital leadership and governmental regulatory bodies (Additional file 4: Figure S4). They also pointed out that clinicians at all levels must share their experiences openly and candidly to identify opportunities for improving patient safety.

Other presenters at the Conference highlighted their experience of communicating with patients and their families around the delivery of care. They noted that early and frequent engagement with patient families reduced the chances of legal actions against a doctor or the hospital (Table 1 and Additional file 5: Figure S5).

**Table 1:** Facilitators and barriers of quality improvement and patient safety [6]

Barriers	Facilitators
Insufficient financial resources.	Organizational and professional development incentives.
Lack of clinical data collection or electronic medical records.	Targeting safety and quality improvement projects to local institutional needs.
Lack of healthcare system familiarity with patient safety.	Local leadership encouragement and support.
Traditional view of medical errors.	Ongoing mentorship and project oversight.
Absence of pre-established patient safety structure.	

Conference panelists also noted that professional training programs can be enhanced to provide physicians with skills that improve their communication with patients and families especially related to the goals and expected outcomes of potential therapies. One approach supported by the experts present at the conference was to inculcate patient-centered communication training in medical, nursing and public health curriculum.

During the conference, representatives from Aviation and Banking industries provided crucial lessons from their own experience. In aviation, the routine use of pre-flight checklists has improved commercial aviation safety by reducing the potential for pilot error. Such checklists are now widely used around the world among all commercial and military pilots. In addition, the banking industries’ long experience in building and maintaining customer relationships provides important examples of how doctors and hospitals can improve their day to day interaction with patients. In healthcare, the use of customer or client focused surveys is becoming widespread in order to identify areas for improving the quality of care and enhancing patient/family satisfaction. Hospitals in Nepal should incorporate such approaches to create effective chains of communication between the patients and a range of professionals at the health facilities.

### Future directions

In Nepal, there is now a critical mass of interest in accelerating the implementation of patient safety programs. Such programs can be implemented in a systematic, step-by-step fashion using limited resources within hospital and clinic settings. Because infectious diseases and antimicrobial resistant pathogens continue to threaten patients, the implementation and dissemination of infection control programs using standardized procedures will provide a strong foundation for building a culture of patient safety. In conjunction with infection control programs that provide regular feedback to hospital physicians and staff, a program for antimicrobial stewardship will be reduce unnecessary antimicrobial usage and will help reduce the spread of multi-drug resistant pathogens. The use of a combination of patient safety indicators related to processes (e.g., regular use of hand hygiene practices by physicians and nurses) as well as patient health outcomes (e.g., reduction in patient deaths or complications) will help provide ongoing information to continuously improve procedures and systems for patient safety. To support patient safety programs, where possible, hospitals will need to carefully consider how housekeeping and environmental services staff can be included as key members of the infection control and patient safety teams. To this end, the creation of a dedicated patient safety committee that regularly meets to review procedures, systems in the hospital will be critical to provide guidance to hospital managers and leadership. Patient safety committees will also be important to pilot test new tools that reduce medical errors in the hospital across all inpatient and outpatient facilities. In Nepal, some hospitals have implemented electronic health record (EHR) systems. While such systems do require a substantial financial investment, they also offer opportunities to improve patient safety by allow physicians and other healthcare staff to better coordinate care, reduce miscommunication around treatment plans and ensure that the patient receives appropriate care in a timely fashion. In Nepal, the use of EHRs has the potential to improve efficiency of patient care and improve follow-up of patients who return to the hospital in future visits.

To support future implementation and evaluation of patient safety programs in Nepal, additional training of physician and staff leadership will be important to help ensure strategic implementation of programs in large and complex healthcare facilities in Nepal. In addition, physician leadership programs will help support the development of multi-disciplinary teams that ultimately take responsibility for implementing comprehensive and rigorous infection control programs as well as other programs for patient safety. Adaptation of programs such as the Henry Ford Health System Physician Leadership Program have the potential to accelerate the introduction of healthcare leadership principles that support staff across all departments in hospitals of Nepal. Based on the participation, energy and enthusiasm shown in the first International Patient Safety Conference in Nepal, the future of patient safety and improved quality of healthcare in Nepal is bright.

## Conclusion

The first international patient safety conference was held in Nepal with the objective of the awareness of patient safety among health professionals and advocacy in developing a comprehensive patient safety framework for the country. The conference emphasized the identification of simple solution with the biggest impact on patient safety and enhancing it. Conference panelists presented the major issues on patient safety and those were medical errors, overstressed medical staff, lack of resources and lack of awareness. The conference recommended on developing of multidisciplinary team for endorsement of patient safety action plan, review and monitoring and formation of accreditation body with safety standards.

## Supplementary information

**Additional file 1: Figure S1.** Panelist responding questions.

**Additional file 2: Figure S2.** Poster presentation session.

**Additional file 3: Figure S3.** Deputy Prime Minister addressing the conference.

**Additional file 4: Figure S4.** Conference delegates and participants.

**Additional file 5: Figure S5.** Presentation from a speaker.

## Data Availability

Not Applicable

## Abbreviations

HER: Electronic Health Record
IPSC: International Patient Safety Conference
UK: United Kingdom
US: United States
WHO: World Health Organization
