# Supplementary figures and images for: Summary of the International Patient Safety Conference, June 28—29, 2019, Kathmandu, Nepal
Source: Patient Saf Surg. 2019 Nov 18;13:36. doi: 10.1186/s13037-019-0214-4 (PMC6862734; doi:10.1186/s13037-019-0214-4)

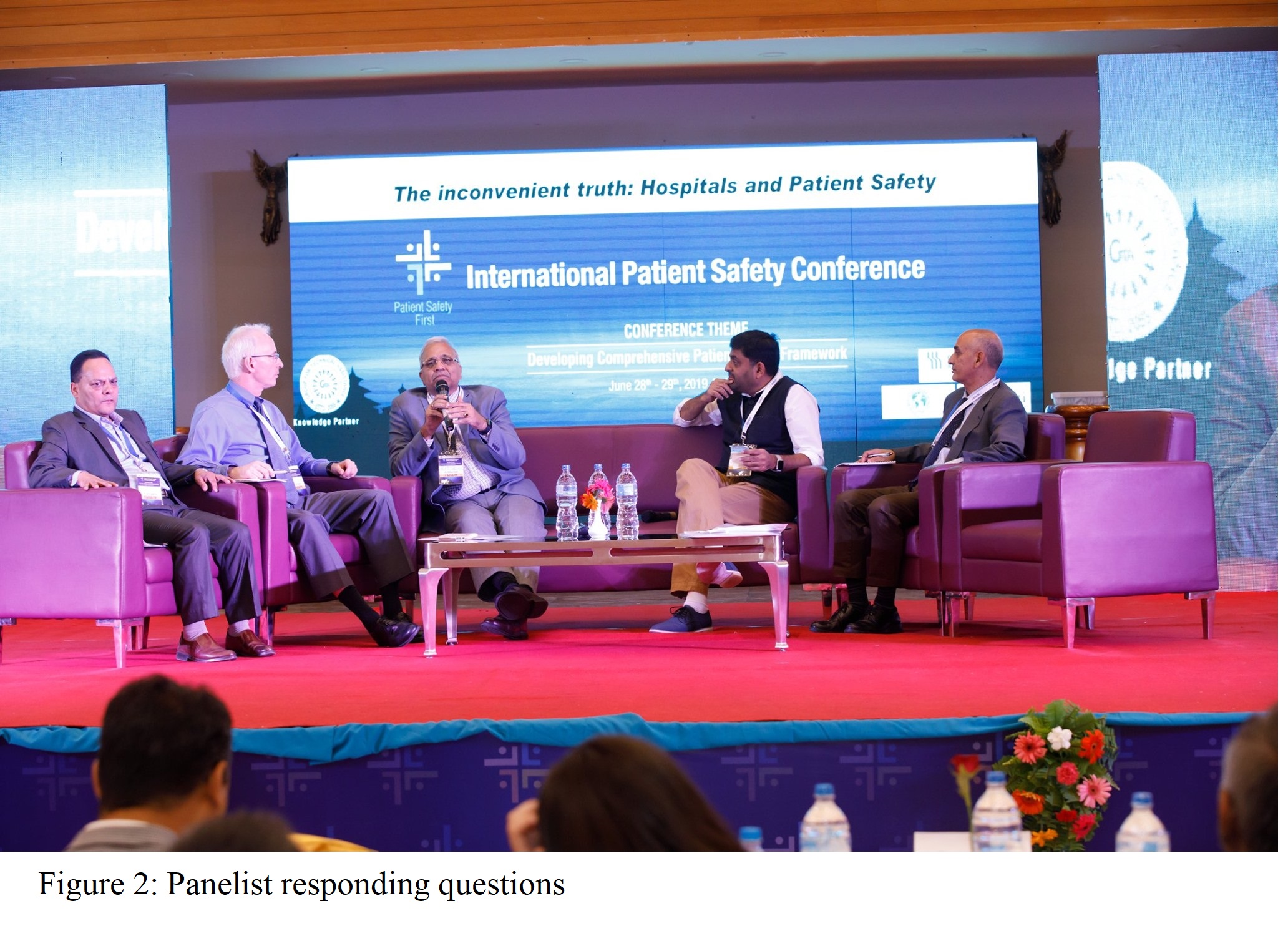

Supplement: Supplementary file 1 — Additional file 1: Figure S1. Panelist responding questions. [file 13037_2019_214_MOESM1_ESM.jpg]

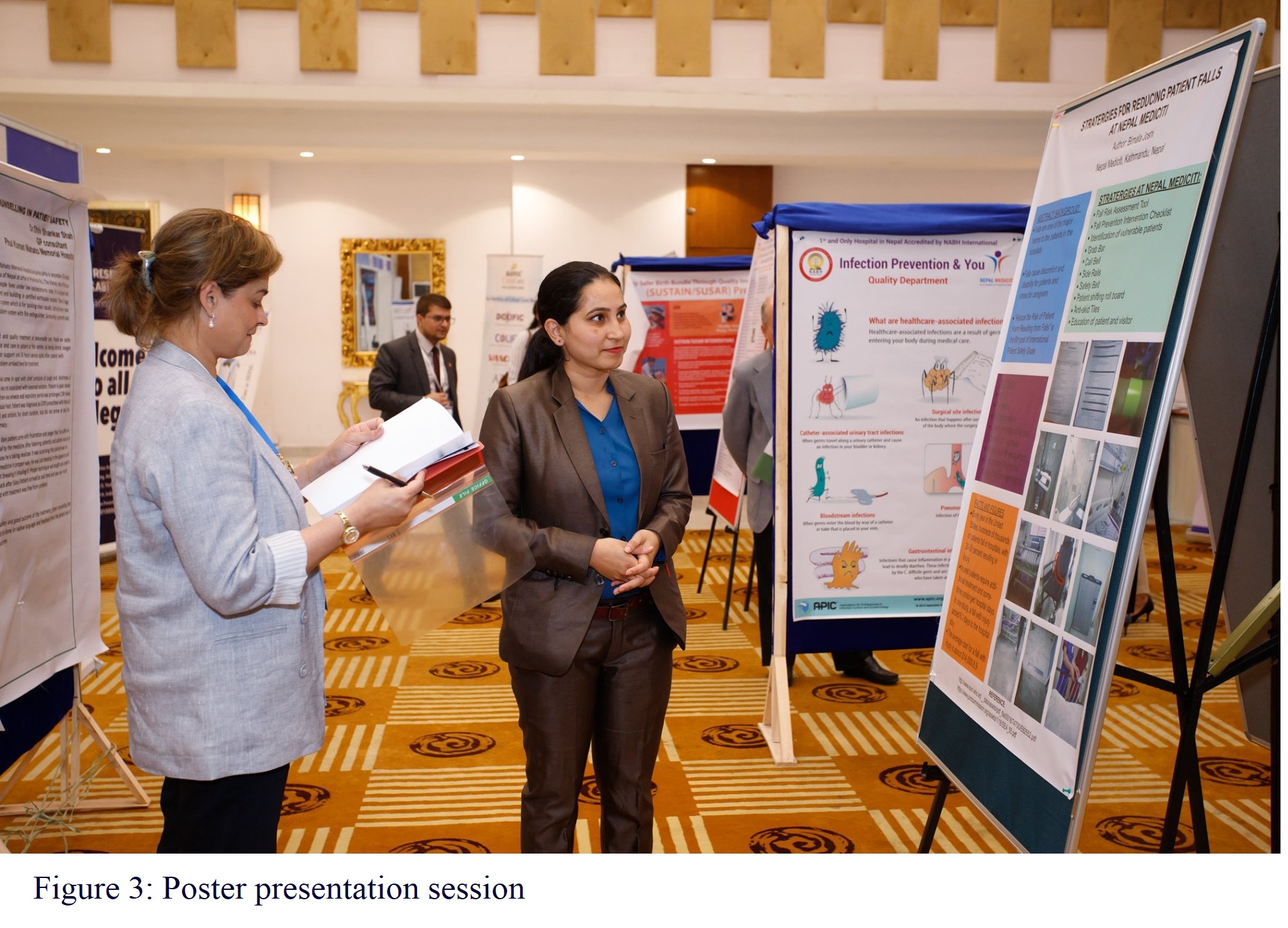

Supplement: Supplementary file 2 — Additional file 2: Figure S2. Poster presentation session. [file 13037_2019_214_MOESM2_ESM.jpg]

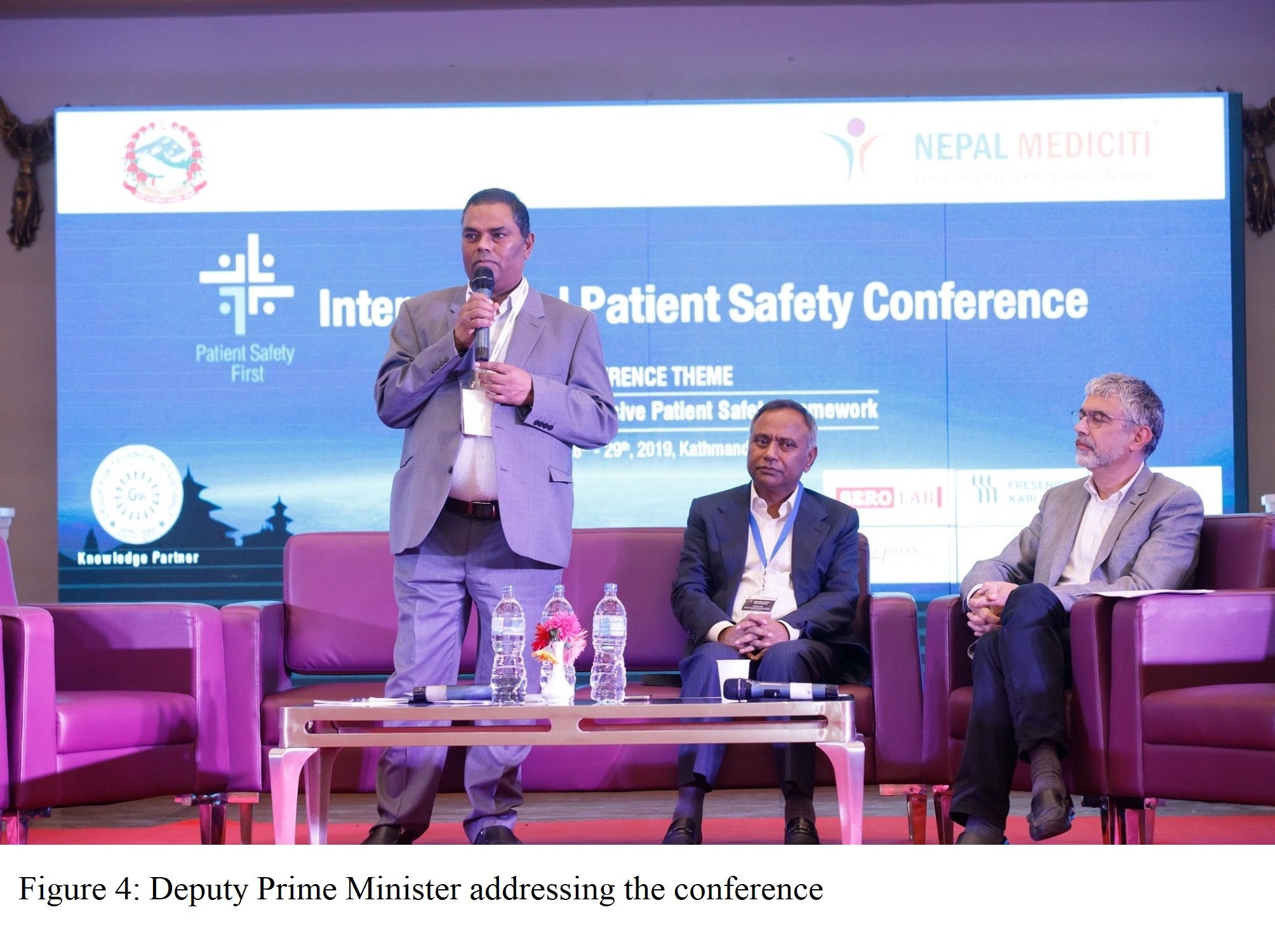

Supplement: Supplementary file 3 — Additional file 3: Figure S3. Deputy Prime Minister addressing the conference. [file 13037_2019_214_MOESM3_ESM.jpg]

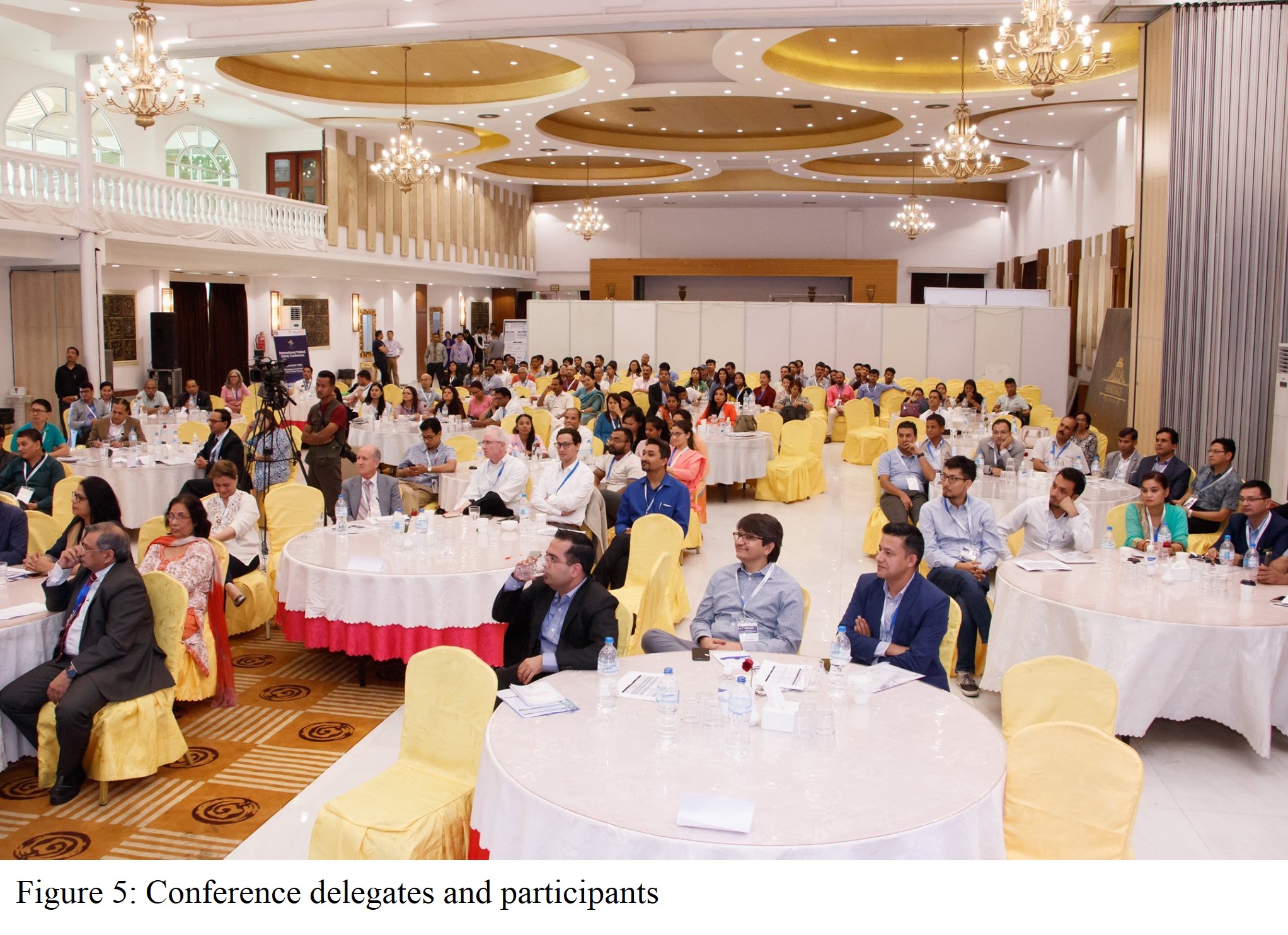

Supplement: Supplementary file 4 — Additional file 4: Figure S4. Conference delegates and participants. [file 13037_2019_214_MOESM4_ESM.jpg]

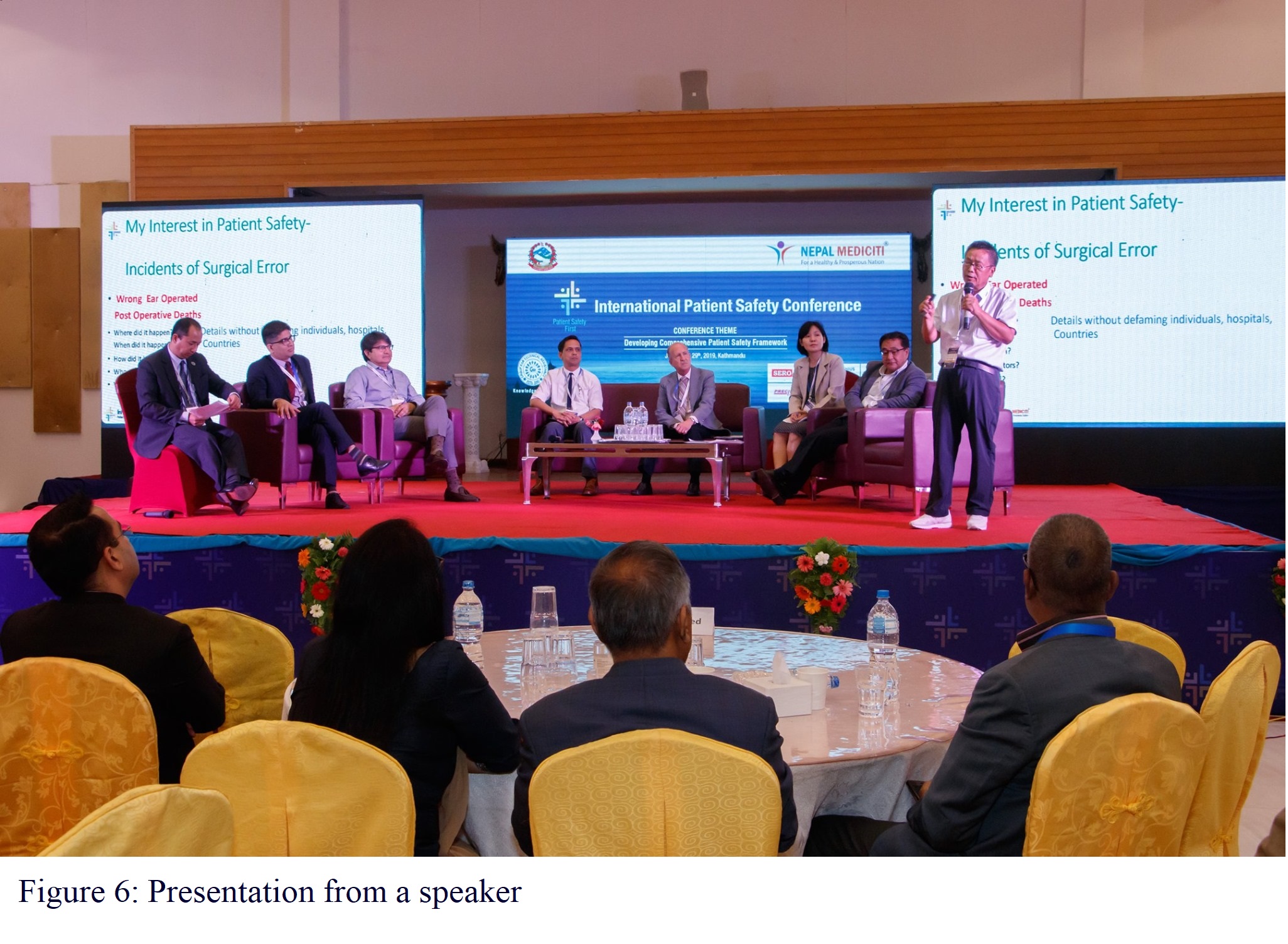

Supplement: Supplementary file 5 — Additional file 5: Figure S5. Presentation from a speaker. [file 13037_2019_214_MOESM5_ESM.jpg]
